# Insulin peptides and their receptors regulate ovarian development and oviposition behavior in *Diaphorina citri*

**DOI:** 10.1111/1744-7917.13048

**Published:** 2022-06-02

**Authors:** Ziye Wang, Delong Tan, Feifeng Wang, Shuhao Guo, Jinhua Liu, Andrew G. S. Cuthbertson, Baoli Qiu, Wen Sang

**Affiliations:** ^1^ Key Laboratory of Bio‐Pesticide Innovation and Application of Guangdong Province South China Agricultural University Guangzhou China; ^2^ Guangdong Laboratory for Lingnan Modern Agriculture Guangzhou China; ^3^ Institute of Facility Agriculture Guangdong Academy of Agricultural Sciences Guangzhou China; ^4^ Natural Medicine Institute of Zhejiang YangShengTang Co. LTD Hangzhou China; ^5^ Independent Science Advisor York UK

**Keywords:** *Diaphorina citri*, insulin‐like peptide, insulin receptor, insulin signaling, reproduction, target of rapamycin

## Abstract

*Diaphorina citri* is an important vector of Citrus Huanglongbing (HLB) disease. After feeding on young host plant shoots, the population of *D. citri* can increase significantly. Females also only lay eggs on young shoots. However, there are few studies on the mechanism of this phenomenon. Exogenous nutrient signals can affect the insulin signaling system of *D. citri* after feeding on young shoots. In this study, the expression of upstream factors *DcILP1*, *DcILP2*, and *DcIR* in the insulin signaling system of *D. citri* was upregulated after feeding on young shoots. After being silenced by RNA interference technology, the results showed that the number of oviposited eggs of *D. citri* was significantly decreased and the ovarian development was inhibited with severe vacuolation. In addition, detection using quantitative reverse transcription–polymerase chain reaction showed that the upstream regulatory gene *DcRheb* of the target of rapamycin (TOR) pathway and the downstream reproduction‐related *DcVg* gene were also significantly downregulated. These results suggest that feeding upon young shoots may upregulate the expression levels of upstream factors *DcILP1*, *DcILP2*, and *DcIR* in the insulin signaling system. The signal will be through upregulating the expression of *DcRheb*, an upstream gene of the TOR signaling pathway. This in turn influences yolk metabolism, which eventually causes the ovaries of female *D. citri* to mature and therefore initiate oviposition behavior.

## Graphical abstract

After female *D. Diaphorina citri* feed on young shoots, *DcILP1*, *DcILP2*, and *DcIR* in the insulin signaling system are upregulated. Interfering with these three genes reduces the expression level of *DcRheb* in the target of rapamycin signaling system and the reproduction‐related gene *DcVg*. This results in varying degrees of stunted ovarian development and a significant decrease in oviposition.

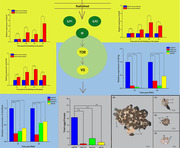

## Introduction

Asian Citrus Psyllid (*Diaphorina citri*) is an important homopteran pest impacting the global citrus industry (Tsai *et al*., 2009; Ruan *et al*., 2012). *D. citri* transmits the devastating citrus disease, Huanglongbing (HLB), which can lead to plant growth retardation and fruit quality degradation that eventually leads to a serious reduction in economic value. *D. citri* can acquire the pathogenic bacteria of HLB, *Candidatus* Liberibacter asiaticus (*C*Las), from infected plants quickly and effectively in its nymphal stages. The resulting *D. citri* adults then disperse and carry the bacteria to new healthy plants through feeding on them. This can lead to widespread distribution of HLB in the field (George *et al*., 2018). In addition, the honeydew secreted by the nymphs of *D. citri* can cause a sooty blotch and so block photosynthesis of the host plant; seriously harming the growth of the citrus crop. At present, no effective bactericides have been found to eliminate HLB, so controlling the vector insect *D. citri* is currently at the core of management programs for HLB.

The insulin and insulin‐like growth factor signaling (IIS) pathway is a conserved signaling pathway found in both vertebrates and invertebrates. This pathway can sense nutritional status and transmit signals through cascades to affect development, metabolism, longevity, and reproduction of the animal. In the IIS pathway, insulin and the insulin receptor (IR), as upstream factors, play an important role in sensing and transmitting nutritional signals. Insect insulin‐like peptides (ILPs) were first isolated from a *Bombyx mori* brain (Wu & Brown, 2006). To date, ILPs have been found in many different insect species with many different functions (Veenstra, 2020). ILPs are upstream factors of the insulin signaling pathway. Molecular studies over the past few decades have shown that ILPs can sense nutritional status through conserved insulin signaling pathways (Wu & Brown, 2006). As an organ related to insect nutrition, the brain of insects also has many insulin‐producing cells secreting ILP. This secretion is regulated by signals produced by the fat body, another key organ regulating nutrient metabolism, similar to the mammalian liver (Delanoue *et al*., 2016). Another important component of the IIS is the IR. This is a transmembrane receptor that triggers the IIS signal transduction cascade and transmits the signal downward after insulin binding (Wei *et al*., 1995; Hubbard, 1997). The IIS regulates various physiological processes such as development, longevity, metabolism, diapause, and female reproduction (Garofalo, 2002). The effect of IIS on reproduction has also been widely studied in various insects, for example, injection of bovine insulin into female adults of *Tribolium castaneum* lacking juvenile hormone (JH) can increase the vitellogenin protein (VG) which is essential for egg maturation and postoviposition embryo development (Wyatt & Davey, 1996; Sheng *et al*., 2011). Furthermore, it has been found that the development of the ovaries of homozygous female *D. Drosophila melanogaster* with dinrE19 mutations is inhibited during the pre‐embryonic stage (Brogiolo *et al*., 2001).

Insect reproduction is known to be affected by two endocrine hormones, JH and 20‐ecdysone (20E) (Bellés & Maestro, 2005). 20E is crucial for vitellogenesis in some hymenopteran, lepidopteran, and dipteran insects, and plays a major role in reproductive processes of mosquitoes and flies; 20E was found to stimulate the expression of VG and oocyte maturation in *Aedes aegypti* (Raikhel *et al*., 2002), and was also found to be involved in ovarian growth and oocyte maturation in *T. castaneum* (Parthasarathy *et al*., 2010). JH also plays a crucial role in the uptake of VG from the ovaries of many insects. In adult female *Pyrrhocoris apterus*, methoprene‐tolerant (Met) is a receptor protein of JH. Deficiency of this receptor severely inhibits the transcription of VG resulting in the VG receptor disrupting the uptake of vitelloprotein into oocytes; this ultimately leads to reduced reproductive capacity (Smykal *et al*., 2014). IIS can intervene in insect reproduction by influencing the content of JH and 20E; these two endocrine pathways being closely related. Female *D. melanogaster* with *DmIR* mutation had impaired ovarian development in the previtellogenesis stage, but treatment with methoprene, a JH analogue, restored vitellogenesis (Tatar *et al*., 2001). In addition, compared with the wild type, the ovaries of *D. melanogaster* with the *DmIR* mutation produced less ecdysone (Tu *et al*., 2002).

In the process of plant development, nitrogen, potassium, and other elements will be transferred from old tissues to new tissues to synthesize characteristic substances in flush shoots and other new tissues. These substances will be ingested by female *D. citri*, so changing their nutritional status after feeding on flush shoots (Sétamou *et al*., 2016) and inducing them to lay eggs. Our previous research demonstrated that the expression levels of JH‐ and 20E‐related genes and VG increase after *D. citri* has fed on flush shoots (Guo *et al*., 2021). The *DcRheb* (Ras‐homologue enriched in brain), *DcRaptor* (regulatory associated protein of mTOR), *DcSin1* (target of rapamycin [TOR] complex 2 subunit MAPKAP1), and *DcS6K* (TOR complex 2 subunit MAPKAP1) genes in the TOR signaling pathway are also all upregulated after mated *D. citri* females have fed on flush shoots (Guo *et al*., 2021). We also previously determined that the synthesis and effect of JH and 20E are closely related to the TOR pathway. The expression of JH‐ and 20E‐related genes was significantly inhibited by interference treatment of *DcRheb*, a key factor in the TOR pathway. These results suggested that feeding on flush shoots induces oviposition in female *D. citri* and is realized by the TOR signaling pathway affecting JH and 20E (Guo *et al*., 2021). However, its upstream pathway has not been fully studied.

The current study therefore investigates the pathways that transmit nutritional signals and seeks to link them to reproduction. Combined with the relationship between the insulin signaling pathway and insect nutrition status as described above, we considered whether the insulin signaling pathway is also involved in reproductive regulation upstream of TOR.

## Materials and methods

### Insects

The *D. citri* used in this study was collected on shrubs of *Murraya paniculata* near South China Agricultural University (SCAU), Guangzhou, China, and subcultured in the laboratory of the Engineering Research Center for Biological Control, Ministry of Education, SCAU. The culturing method was to simply allow the *D. citri* to feed on the host plant *M. paniculata* covered in a white nylon net bag in an incubator at 26 °C, 14 h light and 10 h dark photoperiod, and 60% ± 2% relative humidity (Guo *et al*., 2021).

### Identification of DcILP1, DcILP2, DcIR, and phylogenetic tree construction

For the sake of description, we named the gene XP_008468126 as *DcILP1*, AWT50608.1 as *DcILP2*, and XP_008479213 as *DcIR*. The prepropeptide of *DmILPs* and *DmIR* was identified (Brogiolo *et al*., 2001) and aligned with *DcILP1*, *DcILP2*, and *DcIR* using the clustalx multiple sequence alignment program. A total of 26 ILP amino acid sequences from 16 species and 40 IR from 22 species were used for alignment and phylogenetic tree construction. The prediction of the signal peptide (SP) of *DcILP1*, *DcILP2*, and *DcIR* was made by signalp 4.0. The sequences of the species investigated are listed in the Supporting Information Table S1. The phylogenetic trees were constructed using MEGAX software according to the maximum likelihood method and the bootstrap value was set as 1000. Three‐dimensional models of *DmIR* and *DcIR* were constructed and analyzed using SWISS‐MODEL.

### Expression patterns of insulin key genes DcILP1, DcILP2, and DcIR in Diaphorina citri feeding host

**Fig. 1:**
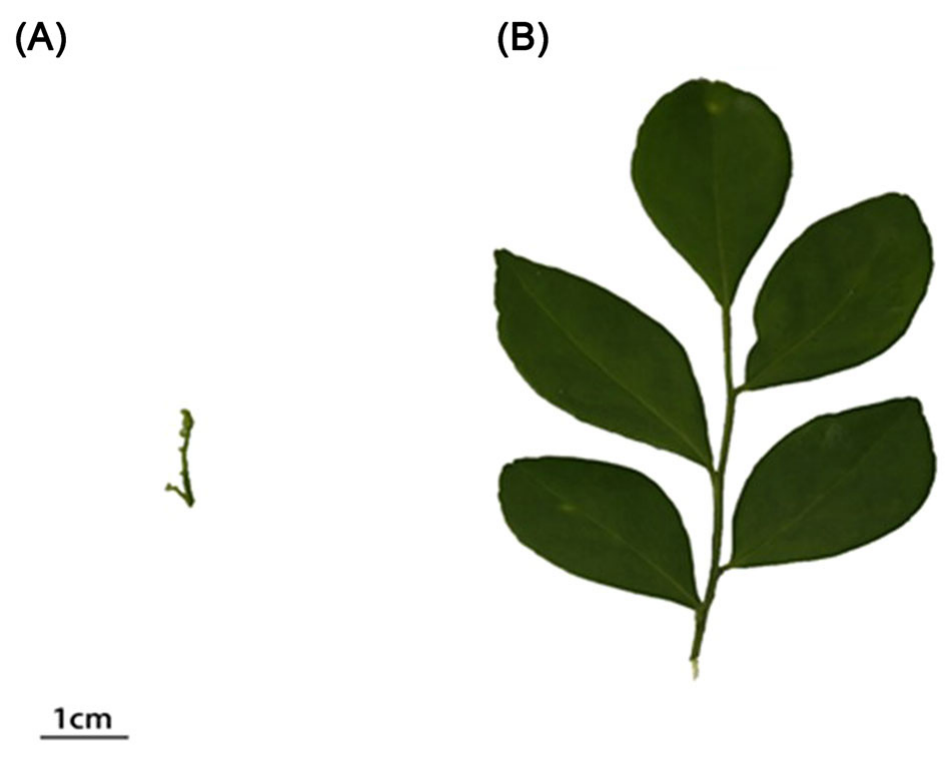
Pictures of flush shoots and mature leaves influencing the reproduction of *Diaphorina citri*. (A) Flush shoots within a week after budding can induce the reproduction of *D. citri*; (B) mature leaves 20 d after budding cannot induce the reproduction of *D. citri*.

Pairings were made according to the ratio of 1 : 2 male to female and placed on mature leaves (20 d after budding) for 7 d. They were then transferred to an environment where only either flush shoots (within a week after budding) or mature leaves were available (Fig. 1). Female *D. citri* were collected at 1, 3, 5, and 7 d after each treatment. Each treatment had three replicates and each replicate had 30 *D. citri*. RNA was extracted from frozen worms using Trizol reagent (Thermo Fisher Scientific Inc. , Waltham, MA, USA) with agarose gel electrophoresis and NanoDrop (Thermo Fisher Scientific Inc.) was used to determine quality and concentration. The cDNA was obtained by reverse transcription of the above RNA samples according to the PrimeScript™ RT Reagent Kit with GDNA Eraser (Perfect Real Time) (TAKARA, Beijing, China). primer 5 software was used to design quantitative reverse transcription–polymerase chain reaction (RT‐qPCR) specific primers for *DcILP1*, *DcILP2*, and *DcIR* genes; *DcActin* gene was used as the internal reference gene (Supporting Information Table S2). The fluorescent dye TB Green Premixture Ex Taq II (TAKARA) was used to detect the expression of *DcILP1*, *DcILP2*, and *DcIR* genes at different time intervals within female adults of *D. citri*. Relative expression was calculated using the 2^−ΔΔCt^ method (Livak & Schmittgen, 2001).

### RNA interference effect of dsRNA on the number of oviposited eggs and ovarian morphology

The dsRNA‐specific primers of *DcILP1*, *DcILP2*, and *DcIR* were designed using primer 5 software (Supporting Information Table S2). The cDNA of the whole *D. citri* was used as a template. The plasmid of enhanced green fluorescent protein (EGFP) was used as the template for the control group (also shown in Supporting Information Table S2). The PCR conditions were 94 °C for 3 min, 40 cycles of 98 °C for 10 s, 55 °C for 30 s, 72 °C for 1 min, and a final step of 72 °C for 10 min. The PCR product was purified by use of a DNA purification kit (Tiagen, Beijing, China). dsRNA was synthesized by the Hiscribe™ T7 in Vitreo Transcription Kit (New England Biolabs Inc., Ipswich, MA, USA).

The *D. citri* were paired according to the ratio of male to female 1 : 2. There were 30 female insects in total. The *D. citri* were placed on mature leaves for 7 d, following which the females were collected for RNA interference (RNAi). The RNAi was performed by diluting dsRNA to 800 ng/μL with RNase‐free water, placing the mated female on ice for a few minutes to cause it to pass out, and then using a 10‐μL Hamilton syringe to locally drop 1 μL dsRNA to the ventral chest between the three pairs of legs. The control was administered with dsEGFP. After being treated with dsRNA, the females of each group were placed on individual shoots and separated by a white nylon net bag. Five biological replicates with 30 females in each replicate were collected on the 1st and 5th days after being placed on the shoots. Total RNA of all samples was extracted and reverse‐transcribed into cDNA, and the silencing efficiency of *DcILP1*, *DcILP2*, and *DcIR* genes after RNAi was detected by RT‐qPCR.

Female *D. citri* mated for 7 d on mature leaves after delivering dsRNA were moved to individual flush shoots, again separated by a white nylon net bag, and moved to new leaves every 3 d to lay eggs. Then, the leaves that had eggs laid on them were cut off, and the number of eggs was counted under a microscope. On day 5 after RNAi, 15 females were randomly selected to dissect their reproductive systems. Photographs were taken with a microscope (Stemi 305, Zeiss, Jena, Germany) using color photography (Axioca 506, Zeiss) using a phosphate‐buffered saline anatomical buffer under a positive fluorescence biological microscope.

### Effects of dsRNA on the expression of TOR signal and reproduction‐related genes

On both day 1 and day 5 after RNAi, 30 females were collected as a replicate, and five biological replicates were collected at each time‐point. Total RNA of all samples was extracted and reverse‐transcribed into cDNA. The RT‐qPCR was used to detect the expression levels of *DcRheb*, a key gene in the TOR pathway, and *DcVg* with *DcActin* gene as an internal reference gene after RNAi (Guo *et al*., 2021).

### Statistical analysis

All data analyses were performed using SPSS 18.0 software (SPSS Inc., Chicago, IL, USA). Gene expression and number of oviposited eggs data were analyzed using Student's *t* test between treatments for mean separation, *P* values less than 0.05 were considered to be statistically significant.

## Results

### Sequence analysis and phylogenetic comparison

**Fig. 2:**
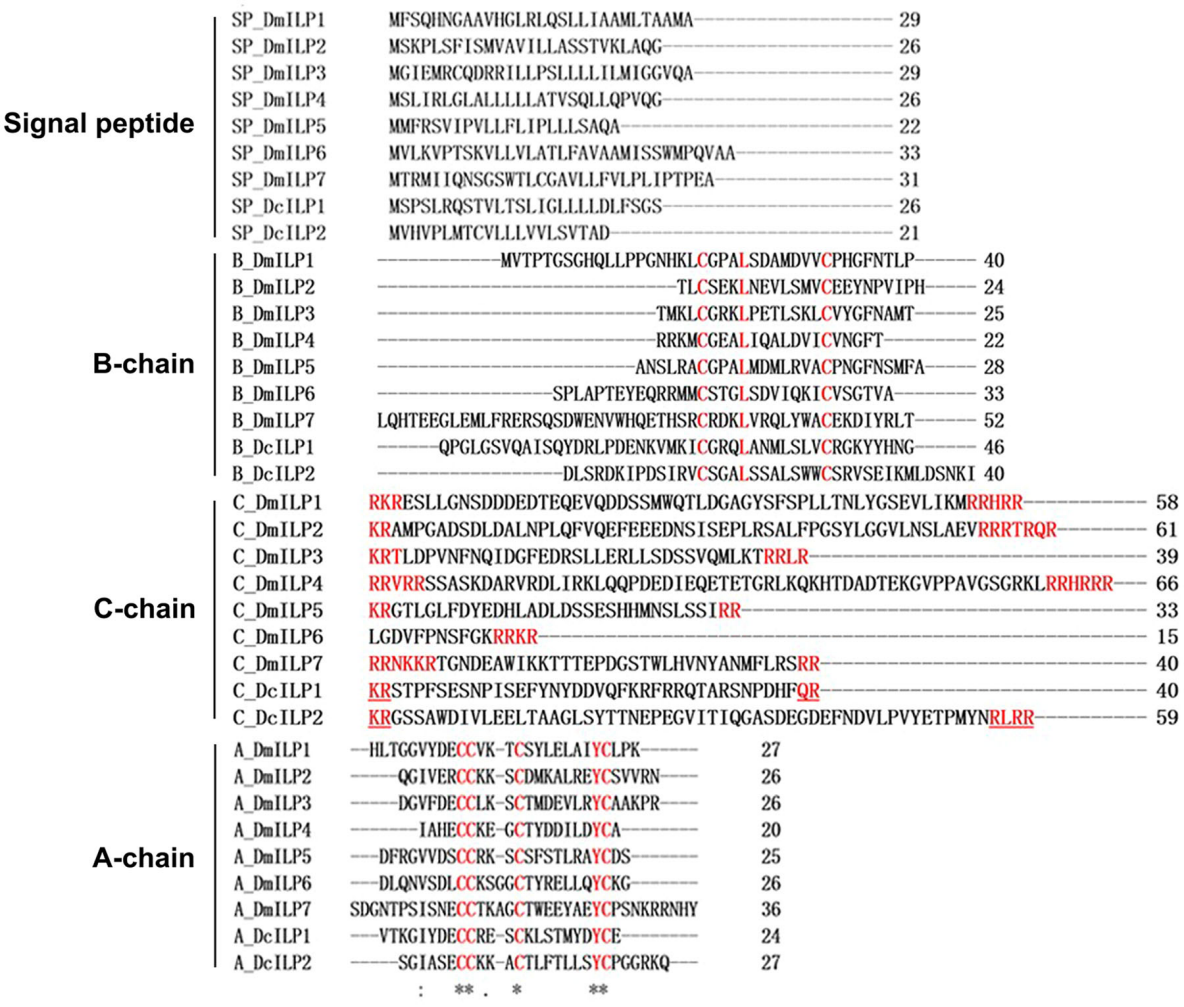
Comparison of amino acid sequences between *Drosophila melanogaster ILP1‐7*, *DcILP1*, and *DcILP2*.

We selected two ILPs that responded to flush shoot feeding and compared the protein sequences of *DcILP1* and *DcILP2* with *DmILP1‐7* of *D. melanogaster* (Fig. 2). The open reading frames of *DcILP1* and *DcILP2* were 411 and 444 bp, encoding 136 and 147 amino acids, respectively. Prepropeptides of *DcILP1* and *DcILP2* also existed of four conserved regions, namely SP, B‐chain, C‐peptide, and A‐chain. We predicted that the SP region of *DcILP1* was located at amino acids 1–26 starting from the N‐terminus, and that the SP region of *DcILP2* was located at amino acids 1–21. SP is followed by B‐chain; the B‐chains of *DcILP1* and *DcILP2* have two conserved cysteines (Cys) separated by 11 other amino acids, and in the fourth position of these 11 amino acids is leucine (Leu), which is highly consistent with *D. melanogaster*. C‐peptide is located behind the B‐chain, and dibasic proteolytic cleavage sites represented by KR, KK, and RR usually exist at the beginning and end. Such sites were also found in *DcILP1* and *DcILP2*. In the A‐chain, *DcILP1* and *DcILP2* both have four Cys and the amino acid before the fourth Cys is tyrosine (TyR), which is a fairly conservative motif and again highly consistent with *D. melanogaster*. Furthermore, the second and third Cys are separated by three amino acids in the A‐chains of *DcILP1* and *DcILP2*. This is also consistent with *ILP1‐5* in *D. melanogaster*.

**Fig. 3:**
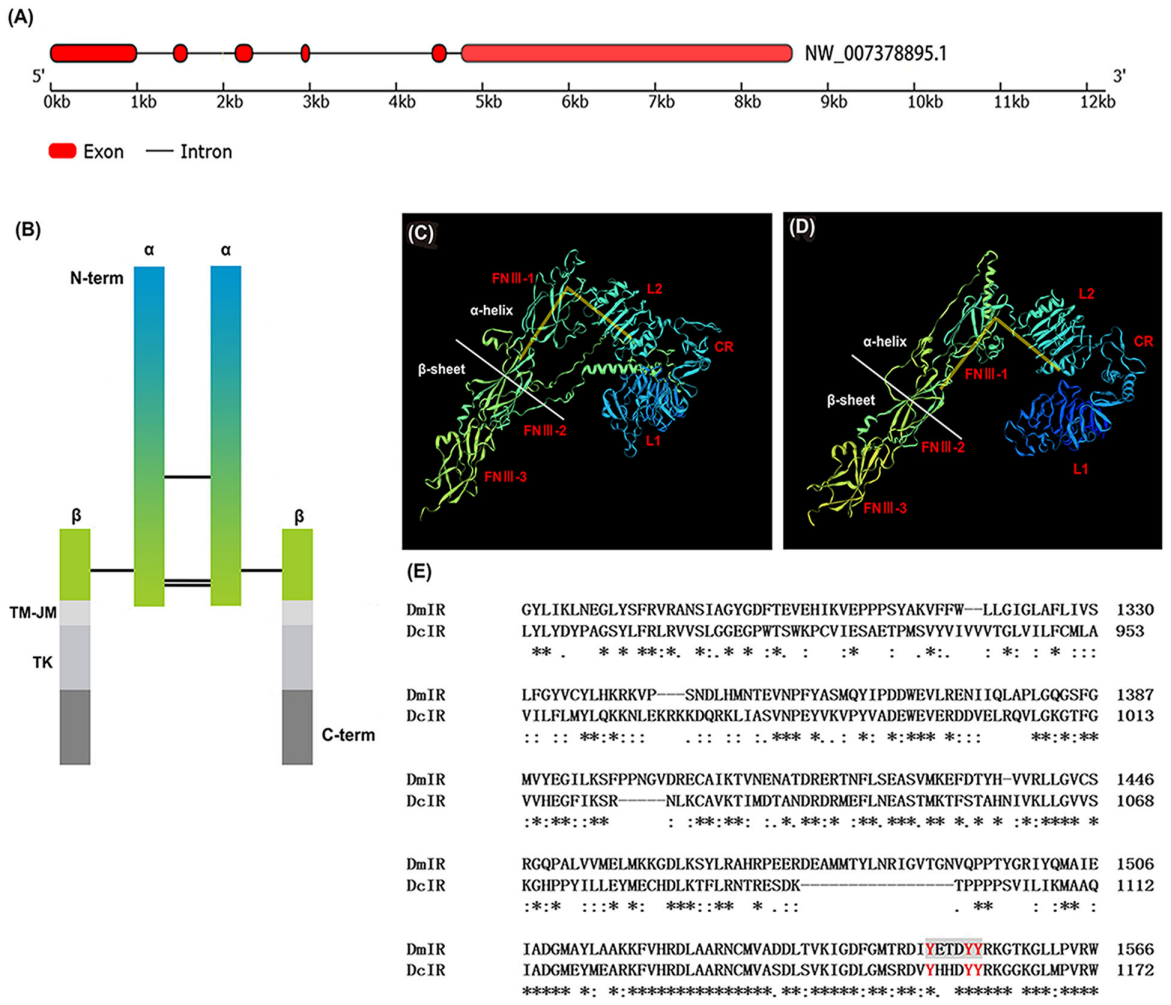
Structure and amino acid sequence analysis of *DcIR*. Three‐dimensional models and comparison of amino acid sequences between *DmIR* and *DcIR*. (A) Introns of *DcIR* sequence; (B) schematic diagram of *DcIR*; (C) tertiary structure of the extracellular domain predicted by *DcIR*; (D) tertiary structure of the extracellular domain predicted by *DmIR*; and (E) comparison of amino acid sequences of intracellular domains between *DmIR* and *DcIR*. Red letters indicate the active region of tyrosine kinase.

The open reading frame of *DcIR* was 4 023 bp in length and encoded 1 340 amino acids. It contained five introns (Fig. 3A) and belonged to cluster II according to Smykal's classification method (Smykal *et al*., 2020). The schematic diagram of *DcIR* is shown in Fig. 3(B), and when comparing the tertiary structure of the extracellular domain predicted by *DcIR* (Fig. 3C) with that of *D. melanogaster* (Fig. 3D), both can be divided into *α*‐chains rich in *α*‐helices and *β*‐chains rich in *β*‐folds containing many of the same domains. Within this, L1 and L2 represent ligand‐binding loops 1 and 2, CR indicates a cysteine‐rich region, and FNIII‐1, FNIII‐2, and FNIII‐3 represent the three fibronectin type III domains. In addition, they can both form a similar “V” structure (marked in yellow in Fig. 3C, D). We also compared the amino acid sequences of the intracellular domains of *DcIR* and *DmIR*. Here, we found that the active region, namely the tyrosine kinase domain of *DcIR* and *DmIR*, was highly consistent, and both had the conserved sequence “motif” (Fig. 3E).

**Fig. 4:**
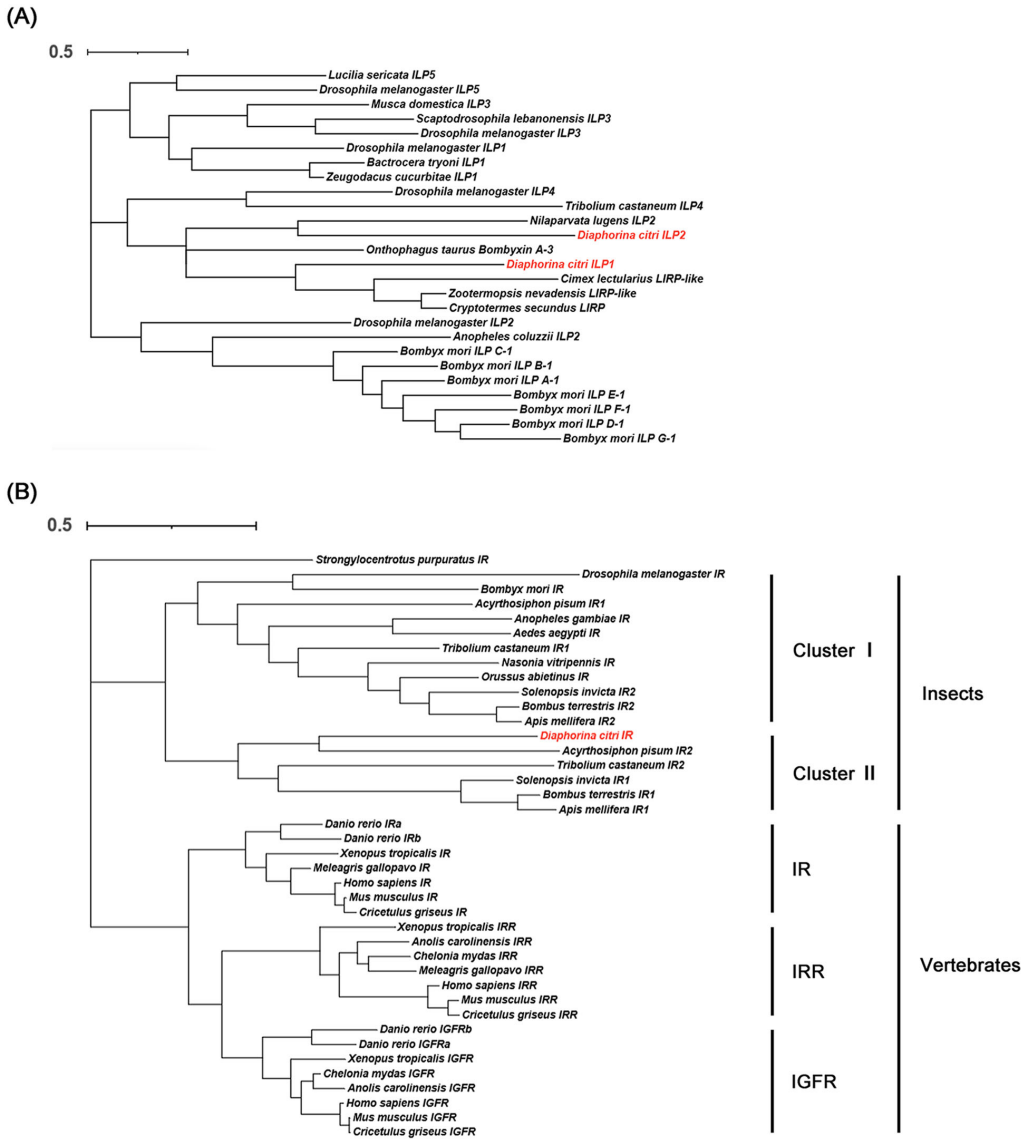
Phylogenetic tree of the insulin like peptides and insulin receptors. (A) Insulin‐like peptides; (B) insulin receptors.

In order to understand the evolutionary relationship between ILP and IR in vertebrates and invertebrates, phylogenetic trees of ILP and IR were constructed. We analyzed 27 ILP amino acid sequences from 16 species and 41 IR from 22 species, these species included vertebrates and invertebrates. For ILPs, insulin‐signaling ligands in vertebrates can be classified into insulin and insulin like growth factor (IGF), which have different functions. Various types of ILPs usually exist in insects. We compared the ILPs of several different insects against DcILPs. The results showed that DcILP1 is clustered with a variety of insect Locusta insulin‐related peptides (LIRP) and it has high homology with LIRP of *Cimex lectularius*, *Zootermopsis nevadensis*, and *Cryptotermes secundus*. DcILP2 has high homology with ILP2 in *Nilaparvata lugens* (Fig. 4A). IR were found in both vertebrates and invertebrates, indicating that ILP and IR can be traced back to at least before the differentiation of vertebrates and invertebrates, and play an important role in some basic physiological activities of organisms. IRs in vertebrates can be divided into IRs, IRRs, and IGFRs groups. Most insects contain only one kind of IR. According to Smykal's classification criteria, cluster I includes the IR1 of *T. castaneum* and the IR of *D. melanogaster*, and cluster II includes IR2 of *T. castaneum*, IR2 of *Acyrthosiphon pisum*, and DcIR (Fig. 4B).

### Expression patterns of DcILP1, DcILP2, and DcIR genes

**Fig. 5:**
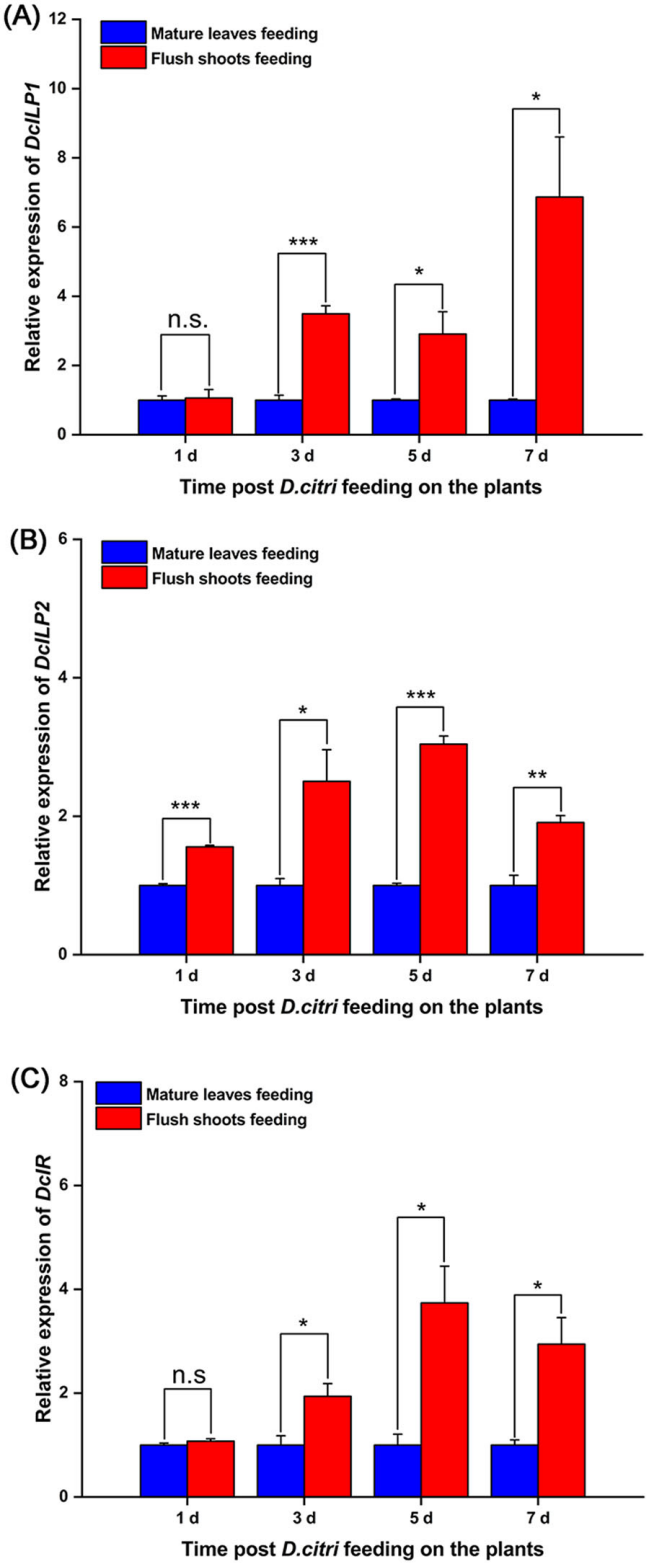
Expression profiles of *DcILP1*, *DcILP2*, and *DcIR* after feeding on flush shoots and mature leaves. (A) Expression profiles of *DcILP1*; (B) expression profiles of *DcILP2*; and (C) expression profiles of *DcIR*. Values represent means ± SE. Gene expression data were analyzed using Student's *t‐* test. The asterisks denote statistically significant differences compared with control (*P < 0.05, **P < 0.01, and ***P < 0.001). n.s. denotes no statistically significant difference compared with control.

In order to study the expression of *DcILP1*, *DcILP2*, and *DcIR* at different times, the mated females of *D. citri* at 1, 3, 5, and 7 d after feeding on either flush shoots or mature leaves were collected for RNA extraction. The RT‐qPCR results showed that the expression levels of *DcILP1*, *DcILP2*, and *DcIR* generally increased when flush shoots were eaten compared with the mature leaves. For *DcILP1*, the expression levels were not significantly different between the two groups at 1 d. However, they were significantly different at 3, 5, and 7 d. The expression levels of *DcILP1* fed on flush shoots were 3.49, 2.91, and 6.87 times higher than those fed on mature leaves at 3, 5, and 7 d, respectively (Fig. 5A). The expression levels of *DcILP2* were all significantly different between the two groups at 1, 3, 5, and 7 d after feeding. The expression levels in the group fed on flush shoots were 1.56, 2.50, 3.04, and 1.91 times higher than those groups fed on mature leaves at 1, 3, 5, and 7 d, respectively (Fig. 5B). For *DcIR*, the expression levels were not significantly different between the two groups at 1 d but were significantly different at 3, 5, and 7 d. The expression levels of *DcIR* fed on flush shoots were 1.94, 3.74, and 2.94 times higher than those fed on mature leaves at 3, 5, and 7 d, respectively (Fig. 5C).

### DcILP1, DcILP2, and DcIR genes RNAi and reproduction

**Fig. 6:**
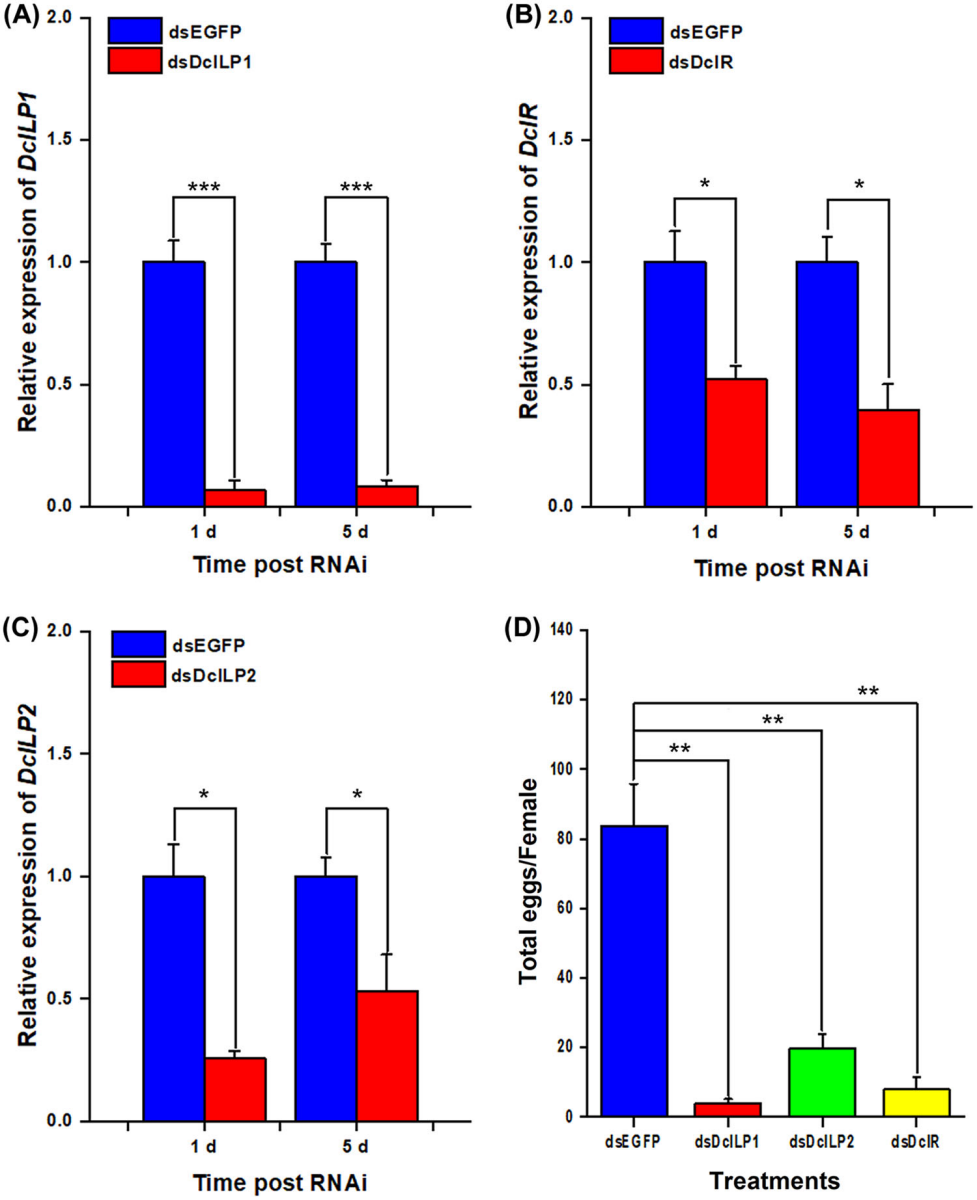
Analysis of fecundity and relative gene expression of *Diaphorina citri* after dsDcILP1, dsDcILP2, and dsDcIR treatment. (A) *DcILP1* RNAi efficiency after dsDcILP1 and dsGFP treatment; (B) *DcILP2* RNAi efficiency after dsDcILP2 and dsGFP treatment; (C) dsDcIR RNAi efficiency after dsDcILP1 and dsGFP treatment; and (D) fecundity of mated female *D. citri*. Values represent means ± SE. Gene expression data and number of oviposited eggs were analyzed using Student's *t‐* test. The asterisks denote statistically significant differences compared with control (*P < 0.05, **P < 0.01, and ***P < 0.001). n.s. denotes no statistically significant difference compared with control.

In order to study the effect of *DcILP1*, *DcILP2*, and *DcIR*, the key genes of the insulin pathway, on the fertility of *D. citri*, *DcILP1*, *DcILP2*, and *DcIR* were suppressed using dsRNA. From day 1 after treatment with dsDcILP1, dsDcILP2, and dsDcIR, RT‐qPCR results showed that the expression levels of *DcILP1* were decreased by 14.62 and 11.92 times (Fig. 6A); *DcILP2* was decreased by 3.89 and 1.88 times (Fig. 6B); and *DcIR* was decreased by 1.91 and 2.52 times, compared with the control group (Fig. 6C). In addition, female *D. citri* were introduced onto flush shoots for feeding after RNAi. Here, the number of eggs in the control group injected with dsEGFP was 83.75 ± 12.09 per female, but in those injected with *DcILP1*, *DcILP2*, and *DcIR* RNAi the insect reproductive ability was almost destroyed with only 4.05 ± 1.11, 19.70 ± 4.20, and 8.00 ± 3.50 eggs per female, respectively (Fig. 6D).

### Effect of silencing DcILP1, DcILP2, and DcIR on ovarian morphology of D. citri

**Fig. 7:**
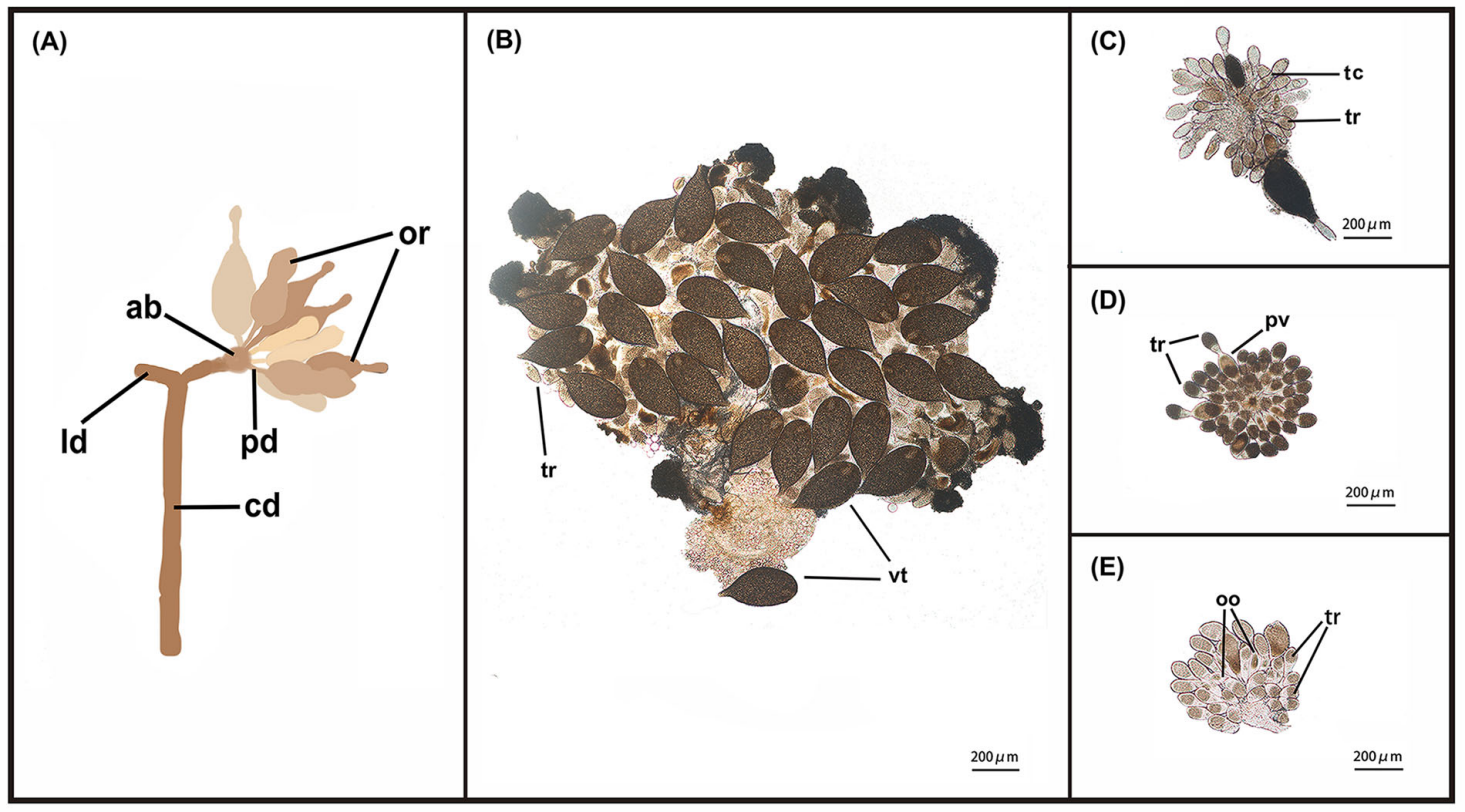
Schematic diagram of partial structure of *Diaphorina citri* ovary and ovarian morphology changes after 5 d following treatment of dsEGFP, dsDcILP1, dsDcILP2, and dsDcIR. (A) Schematic diagram of partial structure of *D. citri* ovary; (B) ovarian morphology 5 d after dsEGFP treatment; (C) ovarian morphology 5 d after dsDcILP1 treatment; (D) ovarian morphology 5 d after dsDcILP2 treatment; and (E) ovarian morphology 5 d after dsIR treatment. ab, apical bulb; cd, common oviduct; ld, lateral oviduct; oo, growing oocyte; or, ovarioles; pd, pedicel; pv, previtellogenic stage oocytes; tc, trophic chord; tr, tropharium; vt, vitellogenic stage oocytes.

The ovaries of *D. citri* are inserted into the apical bulb by a pedicel and connected to the common oviduct through the lateral oviduct (Fig. 7A) (Dossi & Consoli, 2014). We examined ovary development after different treatments. After the mated female adults were introduced onto the flush shoots for 5 d, the ovaries in the control group that were injected with dsEGFP became fully developed. Vitellogenic stage oocytes could also be observed; the obvious characteristics of oocytes in this stage are large and plump (Fig. 7B). However, ovarian development was impaired to varying degrees in the dsDcILP1, dsDcILP2, and dsDcIR groups. For dsDcILP1, 5 d after RNAi, most oocytes were still at the previtellogenic stage, we could observe a large number of trophariums and fewer growing oocytes (Fig. 7C); for dsDcILP2, although there were no vitellogenic stage oocytes, more trophariums could be observed and more previtellogenic stage oocytes were clearly observed compared with dsDcILP1 treatment (Fig. 7D). Five days after dsDcIR treatment, the development of ovarioles was still inhibited compared with that of the control group; there were no vitellogenic stage oocytes but we could observe growing oocytes (Fig. 7E).

### Effect of silencing DcILP1, DcILP2, and DcIR on TOR signal and reproduction‐related genes

**Fig. 8:**
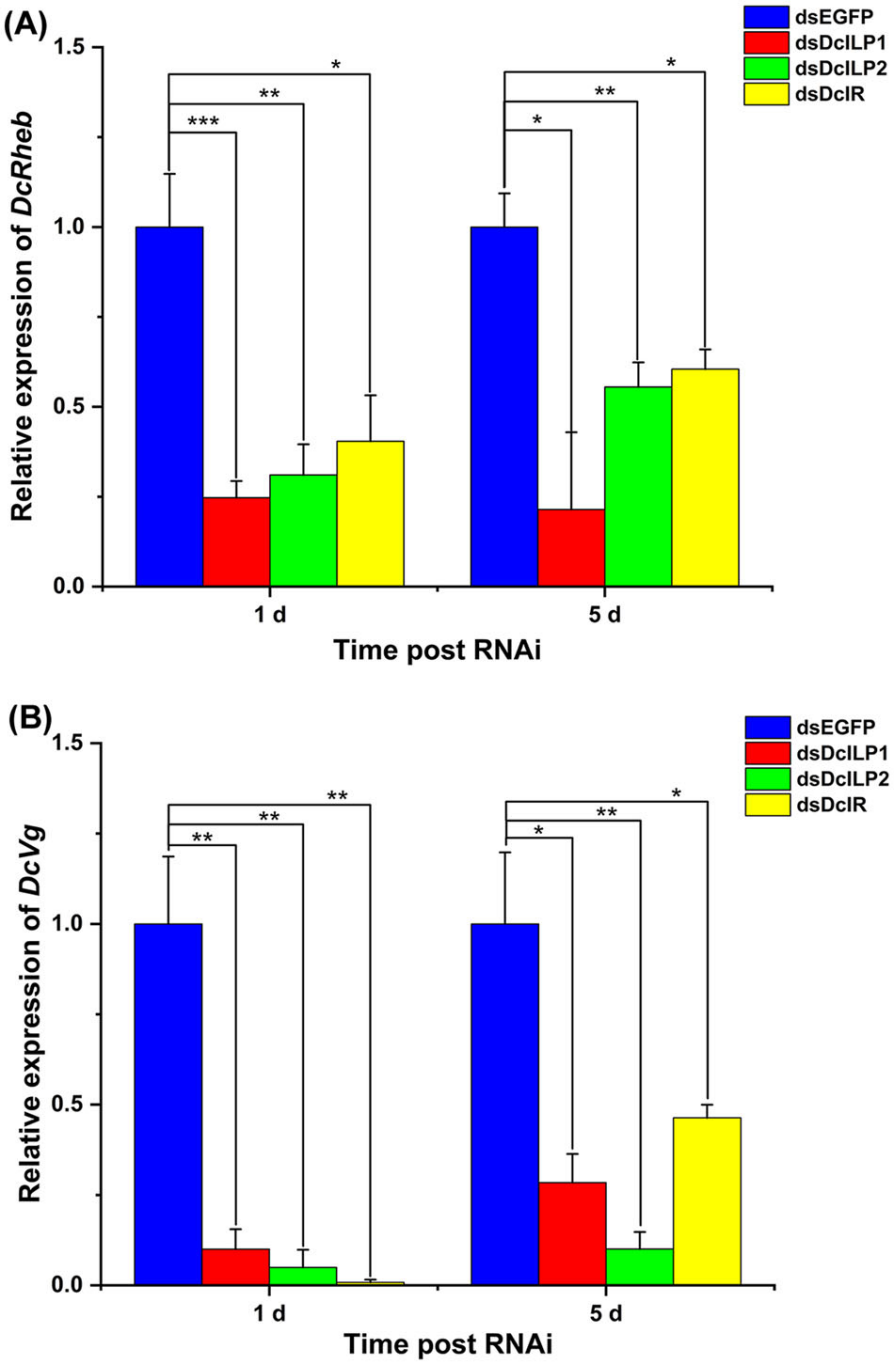
The expression levels of *DcRheb* and *DcVg* after dsDcILP1, dsDcILP2, and dsDcIR treatment. (A) *DcRheb* expression of female *D. citri* after dsDcILP1, dsDcILP2, and dsDcIR treatment for 1 and 5 d; (B) *DcVg* expression of female *D. citri* after dsDcILP1, dsDcILP2, and dsDcIR treatment for 1 and 5 d. Values represent means ± SE. The asterisks denote statistically significant difference compared with control (*P < 0.05, **P < 0.01, and ***P < 0.001). n.s. denotes no statistically significant difference compared with control.

To determine whether the insulin signaling pathway plays an important role in the reproduction of *D. citri*, *DcILP1*, *DcILP2*, and *DcIR* were suppressed using dsRNA. Expression of *DcRheb* and *DcVg* was analyzed at 1 and 5 d after *DcRheb* RNAi treatment, at which time both genes were significantly downregulated. The expression level of *DcRheb* decreased to 24.73% and 21.46% on the 1st and 5th days after dsDcILP1 treatment, and then following dsDcILP2 treatment reduced to 31.06% and 55.54% on the days 1 and 5, respectively. After dsDcIR treatment it reduced to 40.41% and 60.49% on days 1 and 5, respectively (Fig. 8A). The expression of *DcVg* decreased to 10.02% and 28.41% on days 1 and 5 after dsDcILP1 treatment. Following dsDcILP2 treatment, it decreased to 4.95% and 10.05% on days 1 and 5, and after dsDcIR treatment, it decreased to 0.81% and 46.36% on days 1 and 5, respectively (Fig. 8B).

## Discussion

It has been confirmed by this study that after mated female *D. citri* feed on flush shoots, the substances within the flush shoots are taken in and form a kind of nutritional signal to activate the insulin signaling pathway. Following this, the nutritional signal is transmitted to the TOR signaling pathway, which induces oviposition behavior in *D. citri*.

Insulin, IGF and ILPs as IIS signal ligands exist widely in vertebrates and invertebrates. These signaling molecules play important roles in different developmental stages and tissues of organisms. The human insulin signaling system is complex and well documented. Humans have not only insulin and IGF, but also relaxin and human ILPs (INSL3‐7; INSL3‐7) (Hudson *et al*., 1983; Froesch & Zapf, 1985; Chassin *et al*., 1995; Watanabe *et al*., 2011; Lok *et al*., 2012). Insulin and ILPs have also been found in a large number of invertebrates, such as nematodes and insects. There are multiple types of ILPs in most insects, and the number of ILPs varies from 1 to 38. The secretion and function of ILPs in insects are usually related to nutrition. By analyzing conserved sequences, seven DmILPs with different functions were found in *D. melanogaster*. Among them, DmILP2 regulates cell size and number to affect the size of the organism. Neurosecretory cells (insulin‐producing cells) can autonomously sense circulating glucose and secrete ILP2, ILP3, and ILP5, regulated by factors released by intestinal adipocytes (Nässel & Broeck, 2015). DmILP6 is structurally and functionally similar to IGF in vertebrates (Okamoto *et al*., 2009; Slaidina *et al*., 2009), while DmILP7 is similar to relaxin (Miguel‐Aliaga *et al*., 2008; Yang *et al*., 2008). DcILPs were composed of SP, B‐chain, C‐peptide, and A‐chain sequentially, which were compared with DmILPs. Many amino acid motifs were consistently conserved in them. These indicated that ILPs of *D. citri* may have some similar functions to those in *D. melanogaster* described above. As a downstream molecule of ILP, IR is a transmembrane protein that exists on the cell membrane and can receive signals transmitted by different ILPs and then transmits them downward. It has been confirmed in *D. melanogaster* that IR plays a key role in growth, body size, reproduction, and lifespan (Chen *et al*., 1996; Brogiolo *et al*., 2001; Wu & Brown, 2006). This study also analyzed DcIR and found that the protein sequence structure of DcIR was similar to that of *D. melanogaster*, including not only α‐ and β‐chains, but also the inverted “V” structure, indicating that DmIR is likely to play a similar role to *D. Drosophila* IR.

The IIS has been found to affect reproduction in a variety of insects. Studies on silkworm have shown that the body weight, ovary weight, and egg number of *iGfLp* knockout female silkworm were significantly lower than that of the wild‐type of silkworm (Fujinaga *et al*., 2019). Studies on the role of *INR* genes in the fertility of *N. lugens* have shown that the combined injection of dsINR1 and dsINR2 (dsINRS) simultaneously silences the expression of *NlINR1* and *NlINR2* genes, which can reduce the number of eggs laid by *N. lugens* (Liu *et al*., 2020). In our study, *DcILP1*, *DcILP2*, and *DcIR* genes were silenced. The results showed that the number of spawn decreased significantly and ovarian development was also inhibited. These results suggest that the insulin signaling system plays an important role in the development of the oocytes and indirectly affects their oviposition mechanism, which is also consistent with the above results of silkworm and *N. lugens*.

To make the conclusion more convincing, in addition to the above macrocharacteristics, we also performed further analysis using RT‐qPCR technology. The VG, a precursor of vitelloprotein, is the main source of nutrients for insect reproduction and embryonic development (Saucedo *et al*., 2003), and is involved in terminal follicular growth, follicular epithelial differentiation, oocyte development, and other reproductive processes (Stocker *et al*., 2003). We found that the expression level of *DcVG* decreased significantly on the 1st and 5th day after *DcILP1*, *DcILP2*, and *DcIR* interference. This indicated that these three genes could significantly affect the reproduction of *D. citri* and were located upstream of *DcVg*.

Early studies on the population changes of *D. citri* showed that the population number was positively correlated with the number of flush shoots of the plant and the continuous generation of flush shoots could ensure the high growth rate of the *D. citri* population (Tsai *et al*., 2009). Consistent with the change of population size, the initiation and duration of oviposition behavior of *D. citri* was also closely related to the flush shoots of host plants. It has been found that female *D. citri* tend to choose flush shoots less than 6 mm in length on which to lay eggs, and the maturation of the ovary in female *D. citri* is also closely related to the existence of plant buds (Cifuentes‐Arenas *et al*., 2018).

As it has been established with *D. citri* that spawning occurs only after feeding on flush shoots, we speculated that the insulin signaling pathway, as an important signaling pathway for sensing nutritional status, will be activated after the female *D. citri* feeds on the flush shoots. This is due to some specific substances in the flush shoots. Consistent with this speculation, our results demonstrated that the expression levels of *DcILP1*, *DcILP2*, and *DcIR* were significantly higher in the group fed on flush shoots on the 3rd, 5th, and 7th days after feeding than in the control group, respectively.

After sensing various nutritional signals, as an upstream signaling factor of the insulin signaling pathway, ILPs can bind to the IR (Chen *et al*., 1996), recruit substrate Chico (Bohni *et al*., 1999), and activate phosphoinositol‐3‐kinase (Weinkove *et al*., 1999). Phosphoinositol‐3‐kinase phosphorylates phosphatidylinositol 4,5‐diphosphate (PIP2) to phosphatidylinositol 3,4,5‐triphosphate (PIP3). The resulting increased PIP3 can activate Akt (serine/threonine kinase) which then activates other downstream signaling pathways, which then transmit signals (Hansen *et al*., 2014). The TOR signaling pathway is connected with the insulin signaling pathway. Both of these pathways are nutrient‐sensitive pathways that regulate growth rate (Oldham *et al*., 2000; Brogiolo *et al*., 2001; Britton *et al*., 2002; Ikeya *et al*., 2002; Kim *et al*., 2002; Oldham & Hafen, 2003), so it is usually together called the insulin/TOR signaling pathway (Smykal & Raikhel, 2015). In addition, activated Akt has been identified as a key molecule linking the IIS and TOR pathways. It can inhibit the negative regulatory factors of TOR, thus achieving the positive regulatory effect on TOR. A study of female *A. aegypti* found that blood intake causes the neuroendocrine cells of *A. aegypti* to release ILP3, which then activates the IIS and TOR pathways by phosphorylating Akt, and inactivates glycogen synthase kinase 3, a key factor in the yolk development of *A. aegypti*. These results indicate that IIS can be used as an upstream pathway to affect the downstream reproductive process through perception of nutrition, and it is likely to be transmitted through TOR signaling (Hansen *et al*., 2014). In our previous study, it was found that mated female *D. citri* produce oviposition only after feeding on flush shoots, and this process has been proved to be closely related to the TOR signaling pathway, and the effect of TOR on reproduction is realized by affecting the expression levels of 20E and JH, two hormones closely related to insect reproduction (Guo *et al*., 2021). Here in the current study, after silencing *DcILP1*, *DcILP2*, and *DcIR*, we detected the expression level of *DcRheb*, a key gene upstream of the TOR pathway, to decrease significantly, which indicates that nutrition signals from flush shoots are transmitted downward by the insulin signaling pathway through the TOR signaling pathway. Besides TOR signaling, ILP can also influence the reproductive process through fork head transcription (FOXO) (Sim & Denlinger, 2009). Therefore, insulin/FOXO signaling should also be considered in the reproduction regulation of *D. citri* in the future.

**Fig. 9:**
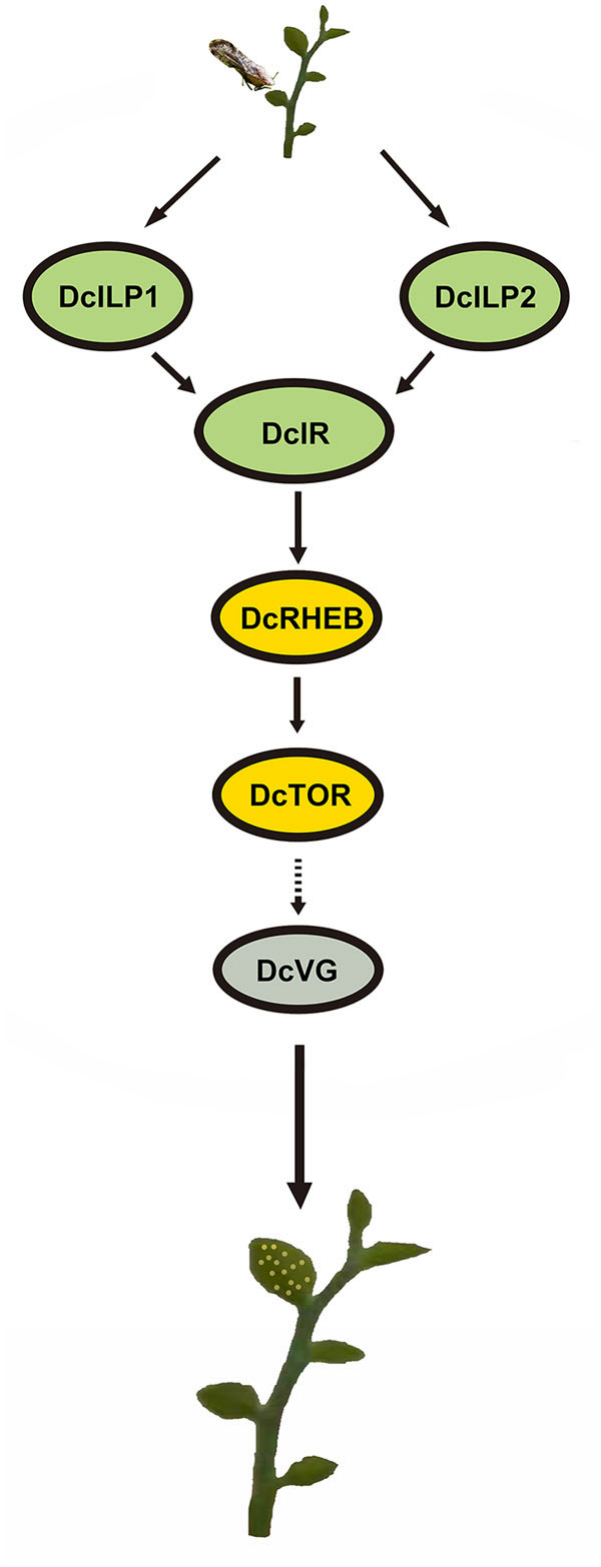
Schematic diagram of pathways related to spawning of *Diaphorina citri* induced by feeding on flush shoots.

In conclusion, after the female *D. citri* feed on the flush shoots, the substances in the flush shoots are taken in and form a kind of nutritional signal to activate the insulin signaling pathway, and the resulting nutritional signal is transmitted to the TOR signaling pathway. An activated TOR pathway leads to an increase in VG expression to induce oviposition behavior in *D. citri* (Fig. 9).

## Disclosure

All authors have seen and agree with the contents of the manuscript and there is no conflict of interest, including specific financial interest and relationships and affiliations relevant to the subject of manuscript.

## Supporting information


**Table S1** Species and accession numbers of ILPs and IRs used for constructing the phylogenetic tree shown in Fig. 4.

**Table S2** Primers used in the study.
