# Supplementary material for: Insulin peptides and their receptors regulate ovarian development and oviposition behavior in Diaphorina citri
Source: Insect Sci. 2022 Jun 2;30(1):95–108. doi: 10.1111/1744-7917.13048 (PMC10084437; doi:10.1111/1744-7917.13048)
Supplement: Supplementary file 1 — Table S1 Species and accession numbers of ILPs and IRs used for constructing the phylogenetic tree shown in Fig. 4. [file INS-30-95-s001.docx]

**Table S1** Species and accession numbers of ILPs and IRs used for constructing the phylogenetic tree shown in Figure 4.

| Species | Accession number | AA | Database | Abbreviation |
| --- | --- | --- | --- | --- |
| *Diaphorina citri* | XP_008468126 | 136 | NCBI | ILP1 |
|  | AWT50608.1 | 147 | NCBI | ILP2 |
| *Drosophila melanogaster* | NP_648359.1 | 154 | NCBI | ILP1 |
|  | NP_524012.1 | 137 | NCBI | ILP2 |
|  | NP_648360.2 | 120 | NCBI | ILP3 |
|  | NP_648361.1 | 134 | NCBI | ILP4 |
|  | NP_996037.2 | 108 | NCBI | ILP5 |
| *Bombyx mori* | XP_004934106.1 | 92 | NCBI | A1 |
|  | NP_001121791.1 | 90 | NCBI | B1 |
|  | NP_001119736.1 | 91 | NCBI | C1 |
|  | NP_001121635.1 | 90 | NCBI | D1 |
|  | NP_001119733.1 | 98 | NCBI | E1 |
|  | NP_001119733.1 | 98 | NCBI | F1 |
|  | NP_001121634.1 | 90 | NCBI | G1 |
| *Nilaparvata lugens* | BAO00958.1 | 136 | NCBI | ILP2 |
| *Lucilia sericata* | XP_037810189 | 118 | NCBI | ILP5 |
| *Onthophagus taurus* | XP_022902688.1 | 112 | NCBI | Bombyxin A-3 |
| *Cimex lectularius* | XP_024084204.1 | 126 | NCBI | LIRP |
| *Cryptotermes secundus* | XP_023701503.1 | 131 | NCBI | LIRP |
| *Zootermopsis nevadensis* | XP_021924502.1 | 132 | NCBI | LIRP-like |
| *Zeugodacus cucurbitae* | XP_011183644.1 | 124 | NCBI | ILP1 |
| *Bactrocera tryoni* | XP_039963704.1 | 121 | NCBI | ILP1 |
| *Anopheles coluzzii* | XP_040238136.1 | 160 | NCBI | ILP2 |
| *Tribolium castaneum* | XP_015840626 | 144 | NCBI | ILP4 |
| *Scaptodrosophila lebanonensis* | XP_030372144.1 | 115 | NCBI | ILP3 |
| *Musca domestica* | XP_005185070.2 | 119 | NCBI | ILP3 |
| *Diaphorina citri* | XP_008479213 | 1340 | NCBI | IR |
| *Homo sapiens* | NP_001073285.1 | 1370 | NCBI | IR |
|  | NP_000866.1 | 1367 | NCBI | IGFR |
|  | NP_055030.1 | 1297 | NCBI | IRR |
| *Cricetulus griseus* | XP_003504238.1 | 1371 | NCBI | IGFR |
|  | XP_003502944.1 | 1301 | NCBI | IRR |
|  | XP_003507789.1 | 1389 | NCBI | IR |
| *Mus musculus* | NP_034698.2 | 1372 | NCBI | IR |
|  | NP_034643.2 | 1369 | NCBI | IGFR |
|  | NP_035962.2 | 1300 | NCBI | IRR |
| *Meleagris gallopavo* | XP_003213352.1 | 1335 | NCBI | IR |
|  | XP_003204644.1 | 1275 | NCBI | IRR |
| *Anolis carolinensis* | XP_003226539.1 | 1335 | NCBI | IGFR |
|  | XP_003228455.1 | 1382 | NCBI | IRR |
| *Chelonia mydas* | EMP31341.1 | 1134 | NCBI | IRR |
|  | EMP41689.1 | 1149 | NCBI | IGFR |
| *Xenopus tropicalis* | XP_002933351.1 | 1362 | NCBI | IGFR |
|  | XP_002939040.1 | 1359 | NCBI | IRR |
|  | XP_002934425.1 | 1240 | NCBI | IR |
| *Danio rerio* | NP_001116701.1 | 1348 | NCBI | IRb |
|  | NP_001136144.1 | 1353 | NCBI | IRa |
|  | NP_694500.1 | 1405 | NCBI | IGFRa |
|  | NP_694501.1 | 1380 | NCBI | IGFRb |
| *Branchiostoma floridae* | XP_002585764.1 | 1336 | NCBI | IR |
| *Aedes aegypti* | XP_001661260.1 | 1371 | NCBI | IR |
| *Anopheles gambiae* | XP_320130.3 | 1318 | NCBI | IR |
| *Apis mellifera* | XP_394771.4 | 1439 | NCBI | IR1 |
|  | NP_001233596.1 | 1690 | NCBI | IR2 |
| *Acyrthosiphon pisum* | XP_001952079.2 | 1486 | NCBI | IR1 |
|  | XP_001942660.2 | 1341 | NCBI | IR2 |
| *Bombyx mori* | NP_001037011.1 | 1455 | NCBI | IR |
| *Bombus terrestris* | XP_003397946.1 | 1415 | NCBI | IR1 |
|  | XP_003393794.1 | 1726 | NCBI | IR2 |
| *Drosophila melanogaster* | AAF55903.2 | 2144 | NCBI | IR |
| *Solenopsis invicta* | ADZ56367.1 | 1432 | NCBI | IR1 |
|  | ADZ56366.1 | 1702 | NCBI | IR2 |
| *Nasonia vitripennis* | XP_003425750.1 | 1759 | NCBI | IR |
| *Tribolium castaneum* | EFA11583.2 | 1394 | NCBI | IR1 |
|  | EFA02828.2 | 1240 | NCBI | IR2 |
| *Strongylocentrotus purpuratus* | XP_784376.3 | 1426 | NCBI | IR |
| *Orussus abietinus* | XP_012280342.1 | 1682 | NCBI | IR |
