# Supplementary material for: Insulin peptides and their receptors regulate ovarian development and oviposition behavior in Diaphorina citri
Source: Insect Sci. 2022 Jun 2;30(1):95–108. doi: 10.1111/1744-7917.13048 (PMC10084437; doi:10.1111/1744-7917.13048)
Supplement: Supplementary file 2 — Table S2 Primers used in the study. [file INS-30-95-s002.docx]

**Table S2** Primers used in the study.

| **Gene** | **Forward primer 5’–3’** | **Reverse primer 5’– 3’** |
| --- | --- | --- |
| **RT-qPCR primers** | | |
| *DcActin* | CCCTGGACTTTGAACAGGAA | CTCGTGGATACCGCAAGATT |
| *DcRheb* | GCTATGATGAAGGAAAACGG | CTAAACCACTGAGGCAGGTAAG |
| *DcVg* | TCACAGCAGCCACCTCACTCA | TATTGGCTGTTCTCCCCAAC |
| *DcILP1* | CAGAGCCAGCAGTCACATTAGG | CCAGGGAGAACTCTGAGAAACG |
| *DcILP2* | CCGAAGGCGTTATCACCA | ACATTCAGAGGCAATACCACTAC |
| *DcIR* | ATGGGAATAGTTGCGAAATAGA | CCCGAGTGAAGCGAAGAG |
| **Primers for dsRNA sythesis** | | |
| dsDcILP1 | TAATACGACTCACTATAGGGGGTGAGCCTGGTGATTGATT | TAATACGACTCACTATAGGGTTGACTCTGAAAACGGGGTA |
| dsDcILP2 | TAATACGACTCACTATAGGGTGCGTCCTTCTTCTAGTAGTC | TAATACGACTCACTATAGGGCCTTCGGGTTCATTGGT |
| dsDcIR | TAATACGACTCACTATAGGGGTTACCAACAAAACCGTCTA | TAATACGACTCACTATAGGGGGTCTGCTGGGAGGATG |
| dsEGFP | TAATACGACTCACTATAGGGGATTAAGTTCAGCGTGTCCG | TAATACGACTCACTATAGGGTTCACCTTGATGCCGTTCT |
